# Local Optimization Strategies in Urban Vehicular Mobility

**DOI:** 10.1371/journal.pone.0143799

**Published:** 2015-12-14

**Authors:** Pierpaolo Mastroianni, Bernardo Monechi, Carlo Liberto, Gaetano Valenti, Vito D. P. Servedio, Vittorio Loreto

**Affiliations:** 1 ENEA, Casaccia Research Center, Via Anguillarese 301, 00123, Rome, Italy; 2 Sapienza University of Rome, Physics Dept., P.le Aldo Moro 2, 00185 Roma, Italy; 3 Institute for Complex Systems (ISC-CNR), Via dei Taurini 19, 00185 Roma, Italy; 4 Institute for Scientific Interchange Foundation, Via Alassio 11/c, 10126, Turin, Italy; 5 SONY-CSL, 6, Rue Amyot, 75005, Paris, France; Beihang University, CHINA

## Abstract

The comprehension of vehicular traffic in urban environments is crucial to achieve a good management of the complex processes arising from people collective motion. Even allowing for the great complexity of human beings, human behavior turns out to be subject to strong constraints—physical, environmental, social, economic—that induce the emergence of common patterns. The observation and understanding of those patterns is key to setup effective strategies to optimize the quality of life in cities while not frustrating the natural need for mobility. In this paper we focus on vehicular mobility with the aim to reveal the underlying patterns and uncover the human strategies determining them. To this end we analyze a large dataset of GPS vehicles tracks collected in the Rome (Italy) district during a month. We demonstrate the existence of a local optimization of travel times that vehicle drivers perform while choosing their journey. This finding is mirrored by two additional important facts, i.e., the observation that the average vehicle velocity increases by increasing the travel length and the emergence of a universal scaling law for the distribution of travel times at fixed traveled length. A simple modeling scheme confirms this scenario opening the way to further predictions.

## Introduction

Nowadays, pervasiveness in the use of ICT (Information and Communication Technology) devices brings the unprecedented possibility of monitoring citizen’s daily activities in urban environments in almost real time [1]. The possibility to access to digital fingerprints of individuals at unprecedented levels of resolution and scale [1, 2] is opening tremendous avenues for a microscopic monitoring and understanding of collective phenomena involving human beings. Despite the clear privacy issues [3, 4] this progress raises, data-driven computational approaches represents a great opportunity for social sciences to become full-fledged experimental sciences [5], a trend recently dubbed as computational social science [6]. The study of human mobility in urban contexts is of utmost importance. With 54% of the world’s population residing in urban areas in 2014 and a growing urbanization trend, both in developed and developing countries, foreseeing an average of 66% of the population to be urban in 2050 [7], mobility in urban areas represents one of the challenging problems of our societies. The understanding of the way people explore the environment they frequent, is indeed crucial to achieve a good management of the complex and problematic processes arising from people collective motion. The solution to issues such as epidemic spreading [8, 9], urban [10] and more in general territorial [11] planning, and sustainable transportation systems [12] cannot abstract from a deep understanding of human behavior in terms of mobility. Therefore, thanks to the recent possibility of monitoring human movements, a new area of interdisciplinary research is rapidly growing, aiming at answering the many questions regarding how people move, their choices and strategies [12]. In this framework, many works have focused on the derivation of universal laws that characterize the diffusion process of individuals from empirical analyses [13–17]. These laws ought to be interpreted as the result of individual strategies [18], the environment in which people move [19, 20] and the complex interactions between them [9, 12–16, 21–25]. Even though each individual manages his/her actions according to free will, human behaviour is anyway constrained by physical, environmental, social and economic factors [21–23]. As a consequence, common schemes and patterns may naturally emerge that could be effectively revealed by a careful statistical approach [21–23].

Human mobility depends both on the spatial and temporal scales of observations as well as on the means of transport adopted, whereas, in the majority of the metropolitan areas around the world, car is still the prevalent transport mode, even for short routes. A physical-based approach has proved to be very useful in order to explain a variety of phenomena on vehicular traffic, arising from the collective motion of cars, mostly in free-ways [26, 27]. These surveys were grounded on Eulerian-like data, meaning that traffic flow is measured at fixed points and at different times. Conversely, with the help of the available modern technology, the individual position of a large number of private vehicles can be dynamically tracked (one refers to *floating car data*), allowing in this way to switch to a Lagrangian point of view, where one focuses on individual tracers and follows their positions in time. In a recent paper [26] it has been shown that the analysis of data of vehicular traffic collected by fixed sensors yields similar results of floating car data so that the latter can be really useful, not only for purely scientific purpose, but also for traffic management.

Since the appearance of this floating car data tracking technology, lots of efforts have been made from an engineering perspective to develop effective methods that would enhance traffic performances [28, 29]. Moreover, a statistical physics approach to urban car mobility based on this kind of data, was recently presented for different Italian cities [21–23, 30], where it was found that simple hypotheses on people’s strategies and the structure of the urban habitat can reproduce the empirical frequency distributions that characterize human travel behaviour. An investigation of car dynamics from a Lagrangian perspective has already been attempted [23] with the result that, for a restricted class of travels [30], the observed dynamics can be retrieved by assuming that the speed time evolution follows a Wiener process [31], so that the accelerations performed by a driver can be described as a sequence of uncorrelated random variables. To the best of our knowledge, the dynamics of car drivers has been described as a diffusion process in which their interactions and knowledge of the city where they move have been disregarded. In this paper, we address the problem of the urban vehicular mobility from a microscopic point of view and we make an attempt to identify the determinants, in terms of the interplay between human strategies and the structure of the urban environment, that underlie the emergence of collective mobility patterns. To this end, we undertake a data-driven perspective and we analyze a large database of GPS tracks of private cars collected in the Rome (Italy) district during the whole month of May 2011. Our empirical findings are consistent with the idea that car drivers’ dynamics is governed by a universal local optimization mechanism. The nature of this mechanism is intrinsically local, in the sense that the globally optimal path cannot be chosen due to lack of knowledge of the environment or to other external conditions. The mechanism is universal due to a scale invariance in the conditional probability of travel time at fixed travel length, suggesting that it occurs in the same way at every travel length scale within the city.

## Materials and Methods

### Data

**Fig 1:**
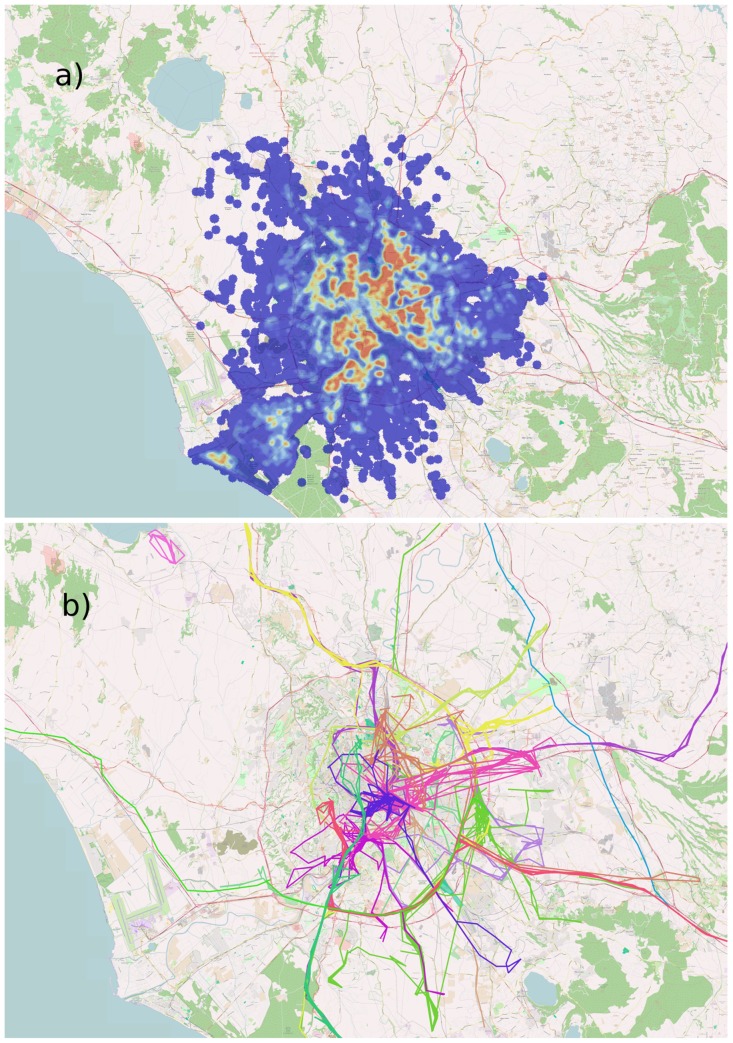
GPS Track Data. (Panel a) Heatmap of the density distribution of the sampling points of the all the trajectories in GPS track dataset. (Panel b) Representation of a small subset of paths coming from the dataset.

Approximately 4% of the whole vehicle population inside the Rome district during the month of May 2011, was monitored on behalf of an insurance company [32]. For that, time, position, velocity and covered distance of single vehicles were recorded by sampling each trajectory at a time scale of 30 seconds (on fast speed roads, e.g., on highways) or at a spatial scale of 2km (elsewhere). This sampling strategy was chosen by that company to ensure a better sampling rate on arterial roads. A further signal was also recorded each time the engine was switched on or off so that a travel is defined as the temporal ordered sequence of points between the engine starts and stops. Precision of space and time measurements is strongly affected by GPS signal quality. The error on time is always around 1s and can be neglected with respect to travel times, whereas the localization error gets at best the value of ±10m. Data can be requested for scientific purposes through the website of the involved company [32]. However in order to ease the reproduction of our findings we provide an aggregated and anonymized version of this data in S1 File. This aggregated dataset does not provide information about the car that performed a certain travel nor the coordinates of the travel itself, but just the length of the travel, the travel time and a timestamp associated with it. In order to analyze ordinary mobility of experienced urban road network users, we select the movements of people only during working days, by considering the same number of the days of the week. Furthermore, we choose to observe only those vehicles whose start and stop positions lie inside the area of Rome municipality, thus most likely focusing on Rome’s inhabitants only. In the end, we observe the travels of 13,527 vehicles during 20 working days in total.Moreover, we consider as a unique travel each of those travels (ca. the 16% of the whole sample) that are composed by tracks containing one or more short stops of 5 minutes, as already established in previous works [21–23]. Changing the value of the 5 minute threshold does not affect much our results. In Fig 1 we show a pictorial representation of the density of the sampled trajectory points in the Rome Urban area, together with a representation of some paths coming from the dataset. Further details can be found in Sec. A of S1 Text.

## Results

### Space-Time Relation

It has already been pointed out how the time spent traveling is deeply connected with the effective energy consumption of human beings so that travel time minimization can be considered to be the driving mechanism in human mobility [18]. For car drivers, such optimization is influenced by the interaction with the complex structure of the urban network and its signature can be spotted in the relation between the space traveled over the network and the amount of time spent by traveling.

**Fig 2:**
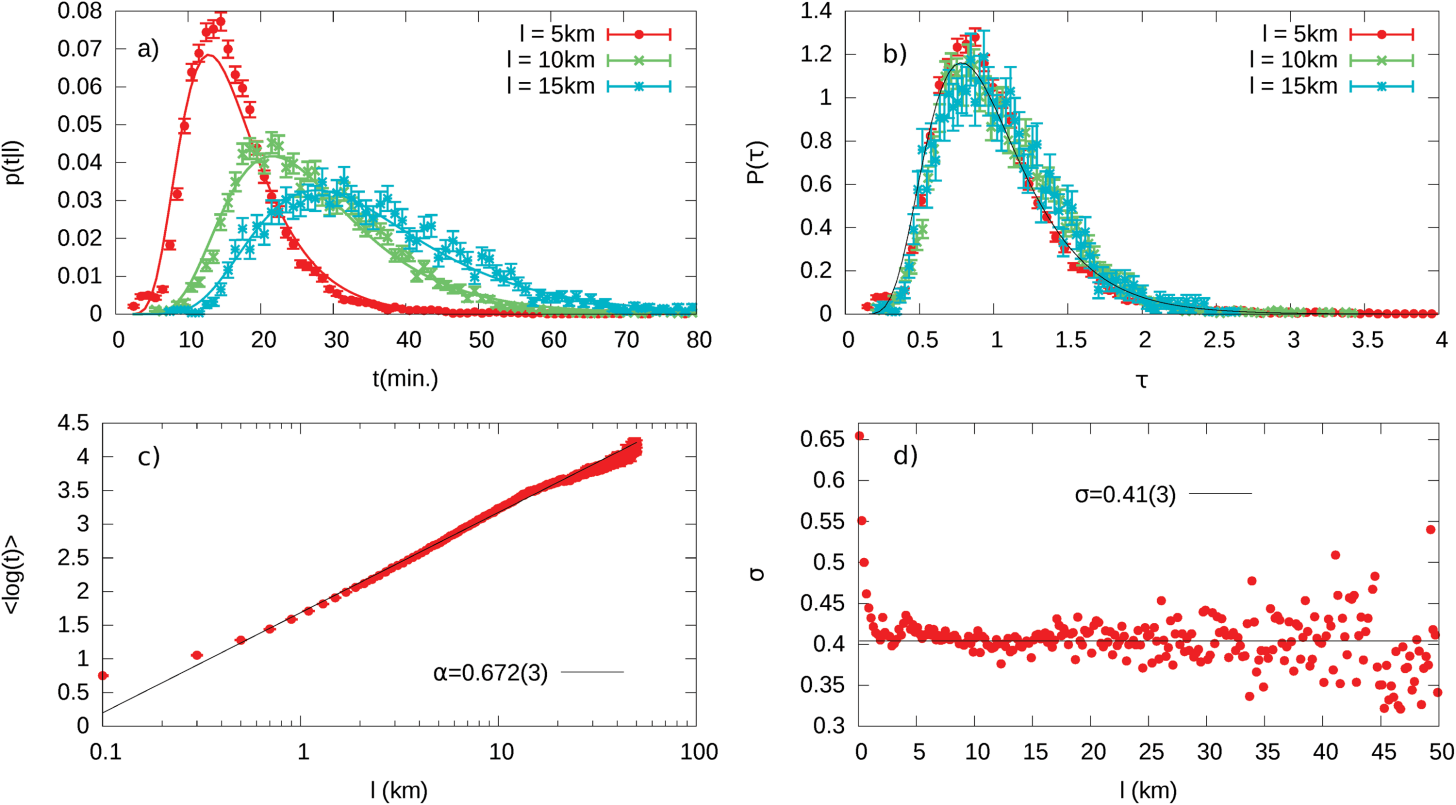
Statistical Properties of Car Driving. (Panel a) Measured distribution *p*(*t*|*l*) of traveling time at fixed travel length for different *l* values. Solid lines refer to the fitted log-normal distributions. (Panel b) Distribution of the scaled variables in correspondence of different values of *l*. (Panel c) Dependence of the experimental mean value 〈log *t*〉 on the travel length *l* (red points). The solid blue line is the best fitting logarithmic function. (Panel d) The standard deviation σ*l* of the variable log *t* inferred from data, as a function of *l*.

For this reason, we start our analysis by looking at the travel length *l* on the road network (i.e., the actual distance traveled) and the travel time *t* (defined as the time elapsed between the start and the stop of the engine—see Methods section) of each recorded GPS track. For each length *l*, we extract the phenomenological frequency distribution *p*(*t*|*l*) of the time *t* needed to travel the length *l*, i.e., we estimate the probability that a travel has a duration of *t* provided that its length has been fixed to *l*. We discover that, regardless of the value of *l*, these distributions can be approximated by log-normal distributions [33] (Fig 2.a):

$$p\left(t|l\right)=\frac{1}{t\sqrt{2\pi \sigma_{l}^{2}}}\exp\left(-\frac{{\left(\text{log}t-\mu_{l}\right)}^{2}}{2\sigma_{l}^{2}}\right)$$ (1)

**Fig 3:**
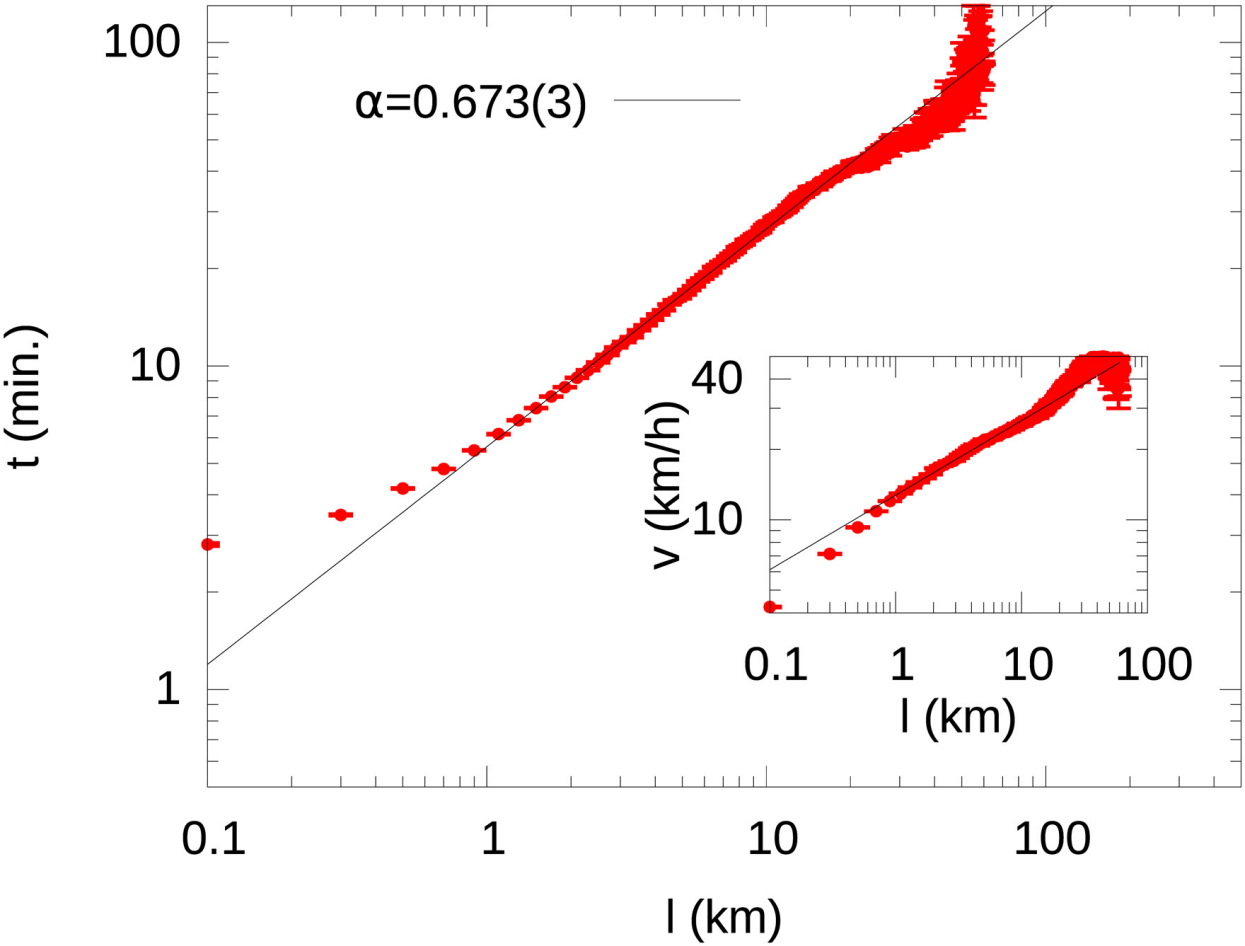
Average travel time dependence on *l* (Optimization Signature). : Experimental average travel time 〈*t*〉 as a function of *l* (red points). The solid blue line is the function 〈*t*〉 = exp(*μ*_*l*_ + *s* ^2^/2) with *μ*_*l*_ = *α*log(*l*/*l*_0_) and *α* and *l*_0_ inferred from the fit in Fig 2.c. This property is the signature of the optimization process performed by car drivers within the urban environment, since it implies a power-law growth in the average speed with the travel length. The inset shows the power-law dependence *v* ∼ *l* ^1−*α*^ of the average velocity *v* on the travel length *l*.

The parameters *μ*_*l*_ and $\sigma_{l}^{2}$ contain the dependence of the distributions on *l* and can be estimated from our dataset by noticing that they represent respectively the average and variance of the log*t* variable at fixed *l*. The parameter *μ*_*l*_ displays a logarithmic growth in *l* as shown in Fig 2.c, i.e., *μ*_*l*_ = 〈log*t*〉 = *α*log(*l*/*l*_0_), with *α* = 0.672(3) and *l*_0_ ≈ 10^−3^m. Note that this relation holds after a transient of small values of *l* until values of about 20km, i.e. before the tail of the sampled path length distribution where we have a worse statistics (see Fig A in S1 Text). Within this interval of length the logarithmic fits data with a $\chi_{\text{red}}^{2}≃1.6$. The logarithmic scale $\sigma_{l}=\sqrt{\sigma_{l}^{2}}$, after a very fast decrease for values of *l* between 0 and 1 km, starts to fluctuate around a constant value *σ*_*l*_ ≃ *s* = 0.41(3) as depicted in Fig 2.c. By substituting these experimental findings into Eq (1) and by relying on the general property of the log-normal distribution [33]$\langle t\rangle =e^{\mu_{l}+\frac{\sigma_{l}^{2}}{2}}∼l^{\alpha },$ we get that the average value of the travel time grows as a power of *l* with exponent *α* ≈ 0.672(3), also confirmed by Fig 3.

The observations above, lead to an effective scaling collapse of the empirical distribution *p*(*t*|*l*) towards the universal function

$$p(\tau )=\frac{1}{s\tau \sqrt{2\pi }}\exp\left(-\frac{{\left(\log\tau +s^{2}/2\right)}^{2}}{2s^{2}}\right)$$ (2)

with *τ* = *t*/〈*t*〉 and *s* = *σ*_*l*_, independently from the travel length *l* (Fig 3.b).

These results are consistent with the hypothesis of a *universal optimization process* somewhat aimed to minimize the total travel time. In particular, the relation between *l* and *t* shown in Fig 3 implies that the average speed *v* = *l*/*t* of a travel grows in a non linear way with *l*, i.e., 〈*v*〉∼*l* ^1−*α*^. Thus, drivers are able to exploit efficiently the surrounding environment when they have to choose their path, and the “efficiency” of their travels (i.e., the ratio between the traveled distance and the amount of time used to go through it—the average velocity) increases with the path length. This process is universal in the sense that the underlying mechanism is independent from the length to be traveled as it is reflected by the scale invariance of the *p*(*t*|*l*) distributions. Interestingly, in Section B of S1 Text we show that this property persists even by aggregating our sample in time slots, indicating that this optimization process is performed independently from the traffic conditions, i.e., from very crowded and congested situations during rush hours to considerably more fluid ones in the late evening.

### Grid model with shortcuts

**Fig 4:**
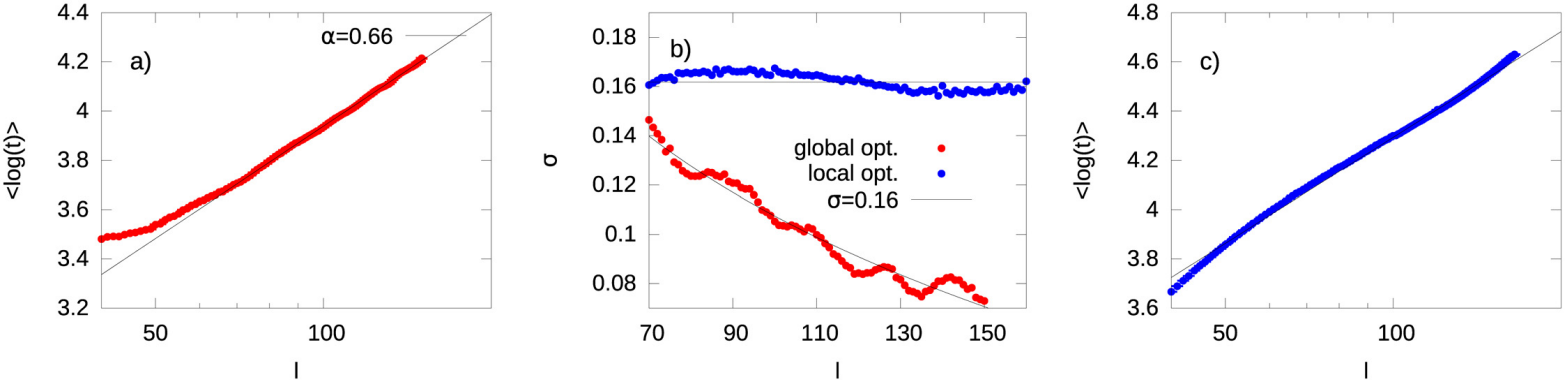
Global Optimization vs Local Optimization in the grid model. (Panel a) Dependence of 〈log *t*〉 on the travel length *l* (red points). The solid blue line is the best fit logarithmic function ($\chi_{\text{red}}^{2}≃2.1$). (Panel b) Dependence of *σ*_*l*_ as a function of *l*. Dots corresponds to paths sampled by using (blue) or not using (red) intermediate optimization steps between the origin and destination nodes of each path. (Panel c) Dependence of 〈log*t*〉 upon travel length *l* (blue points) with paths sampled by picking intermediate stops between the origin and the destination nodes. The solid blue line is the best fit logarithmic function. Both optimization processes yield the correct dependence of *μ*_*l*_ on *l*, but the local piecewise optimization also reproduces the steady behavior of *σ*_*l*_.

**Fig 5:**
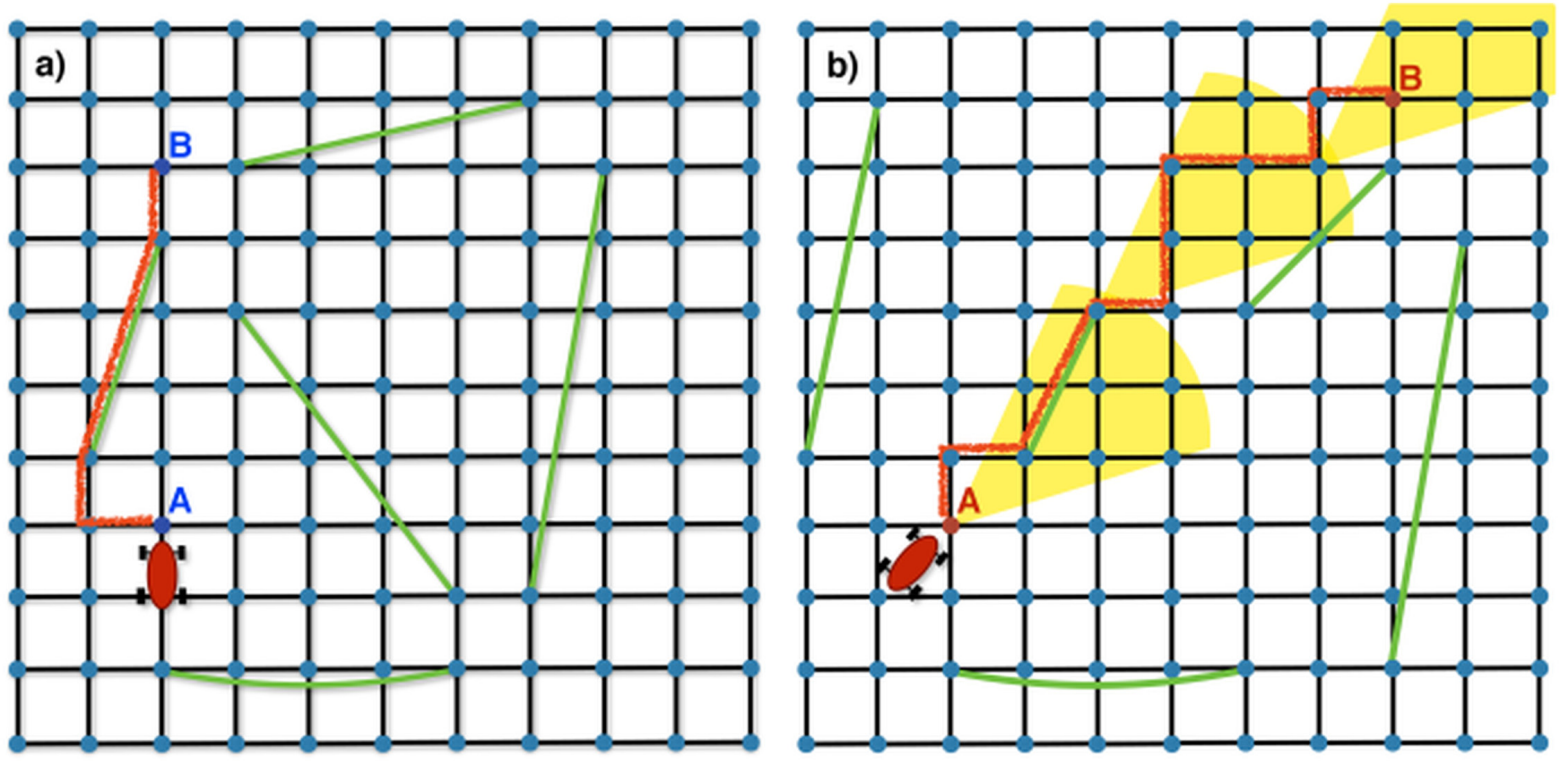
Grid model. (Panel a) Grid model used in the simulations. The black segments stand for ordinary roads of unit length and unit allowed velocity, while the green segments represent high speed connections. Suppose a car has to drive from point A to point B. It may proceed straight on covering five ordinary roads (*l* = 5), or make use of the shortcut by traveling three ordinary and one fast road ($l=3+\sqrt{10}$). In the latter case, depicted in red, the traveled distance is longer but if the allowed velocity on the fast road is sufficiently large, the car would benefit a reduction of the traveled time and choose it. (Panel b) Sketch of the stepwise algorithm used to connect the starting point *A* to the final point *B*. The yellow sectors represent the areas in which the driver can choose his next step. The fast link just below point *B* would be convenient to travel, but it falls outside the yellow sectors and therefore is out of reach.

**Fig 6:**
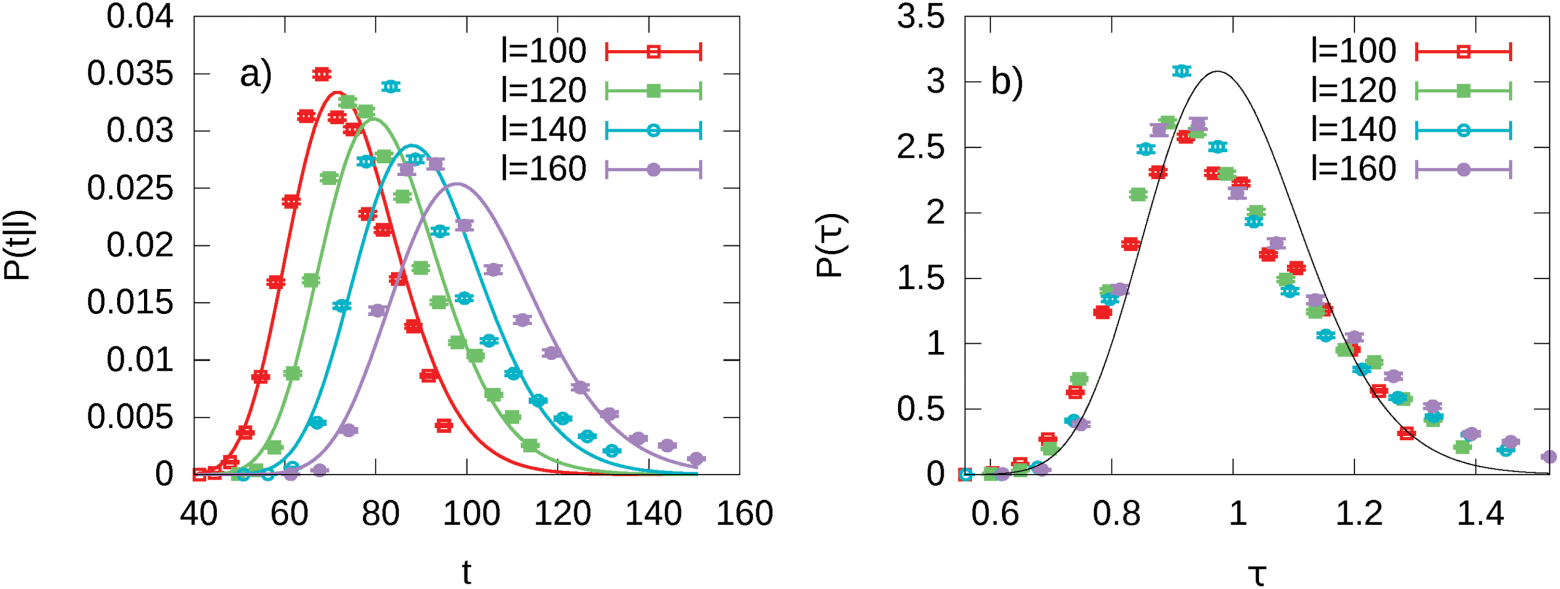
Scaling collapse in the grid model. (Panel a) *p*(*t*|*l*) distribution for some values of path length *l*. Solid lines refers to the fitted log-normal distributions. (Panel b) Distribution of the scaled variable calculated at different values of *l*. The solid black line is the curve in Eq (2). The Kolmogorov-Smirnov test rejects the hypothesis of the universal curve being a log-normal distribution. All the paths are sampled using intermediate steps on a 100 × 100 grid with *N*_shortcut_ = 100 and a fixed speed on each shortcut *v* = 3.0.

The results of the previous analysis emerge from the interplay between the dynamics of the car drivers on the road network and the structure of the network itself. In particular, the non-linear growth of the average car velocity with increasing distance suggests that the drivers try to move in the network in an efficient way and their knowledge of the environment is so that their optimization of the travel time improves as the path becomes longer. Therefore, a first attempt to model their behavior is to assume that, to go from a selected origin to a certain destination on the urban network, drivers are able to follow the path that minimizes their travel time. This assumption allows us to focus solely on the structure of the network. Fast speed arterial roads are crucial for the temporal optimization as the travel distance increases. The practical function of this sort of roads is, in fact, to provide an effective way of reducing the temporal distance between far regions of the city. In a certain sense, this roads resemble the shortcuts of small-world networks [34], with the difference that they have to be traveled with finite velocity, i.e., in finite time. Thus, we propose a proxy of the urban network based on a bi-dimensional square grid with *L* × *L* nodes, where nearest neighbor nodes are connected by unit length links, which can be all traveled with the same unit speed. On top of that, shortcuts are added as links connecting nodes of the grid other than neighboring nodes. The length of shortcuts is assumed to be the euclidean distance between the connected nodes, and their driving speed is set greater than unity. Both normal links and shortcuts are then characterized by a travel time given by the ratio between the length of the link and its associated speed. This assumption on the structure of the road network is in accordance with the results of previous works [35]. In our model we assume that drivers are always able to minimize their travel time, i.e., given a starting and ending point they will follow the shortest path, in terms of travel time. Therefore, to simulate the empirical data, we sample the paths in the synthetic grid network with shortcuts by picking a random departure and a random destination node and by computing numerically the shortest path between them. In order to sample the possible configurations of the grid model, we reassigned the shortcuts every 100 sampled paths. The sub-linear power relation 〈*t*〉∼*l* ^*α*^ is well reproduced by the model (Fig 4.a) with the associated exponent depending both on the density of shortcuts in the network and on their associated velocities. However, in the model, the parameter *σ*_*l*_ clearly decreases as *l* grows (red curve in Fig 4.b), while in reality it should stay constant in average (Fig 3.b). This suggests that, while the short paths are more sensitive to fluctuations, the longer ones are not and the distribution of the travel times defined in Eq (2) becomes sharper for these path lengths. This happens because the optimal trajectories between far nodes tend to be very similar to each other since they involve in average the same number of shortcuts, i.e., they sample the system in a coarse grained fashion and feel a sort of averaged situation. One possibility to recover the observed behavior of *σ*_*l*_ is to argue that the drivers are still able to detect the path with the shortest travel time, but the optimization is not done globally, rather by *intermediate steps*. In our model we can set drivers to come along a series of intermediate points between their travel origin and final destination. The distance between these intermediate points change from one driver to another (it is extracted from a random uniform distribution, see Section D of S1 Text for details) and would reflect their intrinsic ability to optimize the path up to a certain distance. In Fig 5 we depict the two different processes of optimization. This partial optimization mechanism prevents *σ*_*l*_ from decreasing at large values of *l* (Fig 4.c), while preserving the power-law behavior of *μ*_*l*_ (Fig 4.b). Note that the the exponent *α* in both Fig 4.a and 4.c is highly affected by the speed of the shortcuts *v* > 1, but it is not much sensitive to the number of shortcuts itself. With a reasonable choice of *v* (e.g. *v* = 2 or *v* = 3, indicating that on arterials road the speed is twice or three time more than on regular roads), it is possible to obtain a value of *α* close to the empirical one with a very small density of shortcuts, less than a fraction of about 10^−5^ of the all possible shortcuts. See Fig G of S1 Text for details. This in turn, implies that our model reproduces the scaling property of *p*(*t*|*l*) in Eq (2), also observed in the dataset (Fig 6.a and 6.b), although the fitting universal curve does not adhere to a log-normal distribution.

**Fig 7:**
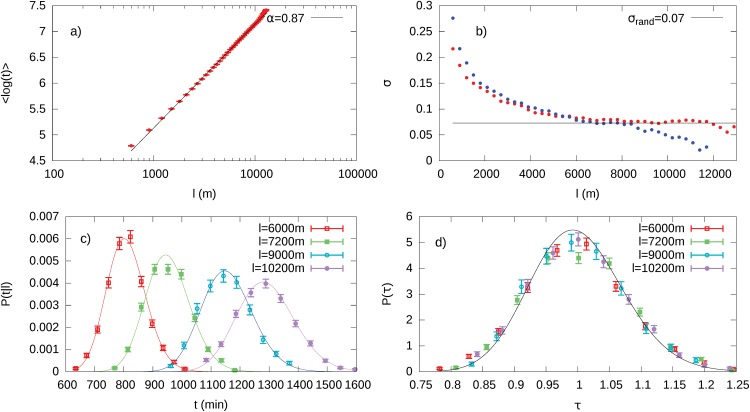
Path Optimization Algorithms on the Rome Urban Network. (Panel a) Dependence of the mean value of the logarithm of travel time *t* upon travel length *l*. These points were obtained using the stepwise optimization algorithm. Solid black line is the best fit with a logarithmic function ($\chi_{\text{red}}^{2}≃3.2$). (Panel b) Standard deviation *σ*_*l*_ of 〈log*t*〉. The paths of these figures are sampled using the navigation algorithm with intermediate steps (red points) and using the shortest path (global optimization) from the origin to the destination (blue points). (Panel c) Distributions *p*(*t*|*l*) for some selected values of *l*. Continuous lines are log-normal approximations of the distributions. (Panel d) Collapse of the same distribution using the scaling property derived in Eq (2). The black curve is the distribution of the scaled variable *τ* = *t*/〈*t*〉.

The proposed grid model with shortcuts, although straightforward, captures the power-law behavior of the average traveling time at fixed distance as well as the universality of the *p*(*t*|*l*) distribution, though not its log-normal shape. One possible refinement of the model would assign to each link an average velocity extracted from a plausible distribution instead of having two fixed values. This refinement would still lack of average velocity correlations between adjacent links. The velocity distribution to employ can be inferred by means of the Google Directions engine [36] and the results are synthesized in Sec. D in Fig J of S1 Text. The result is that by taking into account realistic velocities improves the adherence of the model to the actual picture (the variance *σ*_*l*_ is closer to the real one), but still allows for improvements, being the universal *p*(*t*|*l*) distribution still not well represented by a log-normal distribution. One step forward is to substitute the unrealistic simple grid with shortcuts with the actual urban topology, as we did by considering the cities of Rome and London, so to include correlations between the average velocity of adjacent roads. As a result, Fig 7 proposes again the plots of the quantities shown already in Figs 4 and 6, but now for the Rome Urban Network (more details can be found in Sec. F of S1 Text together with the same results for the London Urban Network). In this latter case, still by considering drivers optimizing their routes stepwise, we get a good quantitative agreement with experimental data. Despite the qualitative agreement, the exponent of the power-law growth of the travel time is higher in our model on realistic networks. Moreover, the constant asymptotic value of the fluctuations is smaller than the empirical one by a factor of two. We argue that this is an effect of the aggregation of different traffic conditions at the level of road segments performed by using the travel time of Google Directions. In fact, while a real-life car will experience all the dynamical fluctuations due to the instantaneous urban traffic, in our approach this effect is averaged before the path is sampled when the average travel time is assigned to a street, resulting in smaller fluctuations and in a smaller improvement of the driving performances with the traveled length. Moreover, this allows us to get some hints on the possible (multiplicative) effects yielding the log-normal *p*(*t*|*l*) distributions (see Sec. G of S1 Text).

As a final remark, in our grid model the number of nodes that can be reached at fixed travel time depends on time as a power-law with exponent larger than 2, as already observed in previous works in case of German cities [35]. In particular, we found that the dimension is larger than 2 also with small numbers of shortcuts and approaches a stable value (depending on the velocity *v* on the shortcuts) as *N*_*shortcuts*_ increases (see Fig H in S1 Text).

## Discussion

We presented an experimental analysis carried on a large database of vehicle mobility in an urban environment. In particular, we focused on the estimated conditional probability *p*(*t*|*l*) that a travel time *t* is needed to reach a fixed distance *l* and showed that with a suitable transformation, it collapses onto a universal curve, reflecting the existence of a universal mechanism. The average time to travel a distance *l* is not linear in *l*, as it would in case of roads traveled at constant average velocity, but shows a sub-linear power-law behavior. Because of that, the average velocity grows as a power-law with the traveled distance. These results are consistent with the idea of an optimization process performed by car drivers in the choice of the path within the city, responsible for the increase of performances as the distance to be traveled grows. To understand the key features at the base of this process taking place on urban road networks, we designed a simple model of driver behavior with stepwise travel time optimization, on a straightforward road network (a two-dimensional grid) with arterial roads (euclidean shortest paths between pair of nodes, traveled with higher speed). Our simple model already captures most of the overall traveling statistics, but leaves space for further improvements. To this aim, we apply the stepwise optimization process to the actual urban network with the realistic average velocity of roads extracted with the Google Directions engine. Doing so, we fully reproduce the overall observed statistics of urban vehicular mobility.

The experimental results are consistent with the idea that this process is not performed with a global vision of the surrounding environment, but is carried out stepwise. Practically, drivers seem to pick up their routes according to a *local optimization criterion*, i.e., they fix some milestones along the path and try to choose the fastest roads that connect these intermediate destinations. In this way, the emerging route is not always the optimal one in terms of travel time, meaning that people’s perception of urban road network is not global in general, i.e., they are not aware of all possible routes, rather just of some of them. In this sense we can suggest that improved info-mobility systems could make drivers conscious of the best global routing choice in order to reduce travel time and conversely fuel consumption with related CO_2_ emissions. The full understanding of the real local optimization process may give useful information on the way the city is perceived by car drivers, leading to a better planning of future urban networks. We remark that studies on this subject might be performed both by relying on historical or future traffic data or by means of social experiments [37].

## Supporting Information

S1 Text Supporting Information Text for this paper. One PDF paper with details on the materials and methods.
(PDF)

S1 File Supporting Information Data. One compressed text file containing aggregated data that can be used to reproduced the findings of this paper. Each row of the text conatins the starting day, starting hour, arrival day, arrival hour, travel time in seconds, travel length in meters of each travel used for the data analysis.
(BZ2)
