# Supplementary material for: Local Optimization Strategies in Urban Vehicular Mobility
Source: PLoS One. 2015 Dec 14;10(12):e0143799. doi: 10.1371/journal.pone.0143799 (PMC4682824; doi:10.1371/journal.pone.0143799)
Supplement: S1 Text — One PDF paper with details on the materials and methods. (PDF) [file pone.0143799.s001.pdf]

Local optimization strategies in urban vehicular mobility

Supporting Information File S1

Pierpaolo Mastroianni[1,2], Bernardo Monechi[2,*], Carlo Liberto[1], Gaetano Valenti[1], Vito D.P. Servedio[3,2], Vittorio Loreto[2,4,5]

1 ENEA, Casaccia Research Center, Via Anguillarese 301, 00123, Rome, Italy
2 Sapienza University of Rome, Physics Dept., P.le Aldo Moro 2, 00185 Roma, Italy
3 Institute for Complex Systems (ISC-CNR), Via dei Taurini 19, 00185 Roma, Italy
4 Institute for Scientific Interchange Foundation, Via Alassio 11/c, 10126, Turin, Italy
5 SONY-CSL, 6, Rue Amyot, 75005, Paris, France
*** Corresponding Author: mone.berna@gmail.com**

A GPS signal quality and data preprocessing

The Global Positioning System (GPS) is based on the reception of radio signals cast by satellites orbiting the Earth. The position of the device is inferred by estimating the distance between the device and a variable number of satellites (the more satellites are connected, the better is the signal quality). Therefore, the precision of GPS position measures depends on the precision in determining distances between the receiver device and satellites. Row data signal quality (SQ) is expressed by an integer ranging from 1 to 3. If $SQ = 1$ the position of the device is determined with less than two satellites and there is a large uncertainty on the position of the vehicle. If $SQ = 2$ two or three satellites are connected, while more than three satellites are visible whenever $SQ = 3$. In this last optimal case the uncertainty on the car position is around 10 m.
A large number of private vehicles (2.5% of the population in the whole Italy, 4% in the Rome district, data of year 2011) is equipped with an On Board Unit (OBU) that possesses three fundamental elements: a GPS locator, an accelerometer for crash detection and a GSM-GPRS module to communicate recorded data to the data service center. The OBU records selected data about car usage. Specifically, a single measure delivers the vehicle position and the time of recording. The OBU itself can estimate, from time and space measurements, the absolute speed value, its direction and the distance traveled on the road network since the previous record. Furthermore, at each position measurement, engine status (ignition, a going, off) and GPS signal quality are recorded.
The data preprocessing operations addressed mostly the travels start and stop definitions. In fact, a signal loss can produce a wrong definition of the beginning or the end of a trip. Each measure includes information on the engine state (ES): $ES = 0$ means that the engine is being switched on while for $ES = 2$ the engine is being switched off. When $ES = 1$ the vehicle is traveling. Therefore, we used the value $ES = 0$ to detect the origin of a trip and $ES = 2$ to spot the destination. In case the values of ES are not available (eg., when parking locations lie underground), we use the first temporal measure of a vehicle as its origin, while the last measure is assumed to be its destination. Furthermore, if between two successive points with $ES = 1$ there is a time lapse larger than 30 min, we consider the first point as the end of a travel and the second one as the beginning of a new trip. Finally, we filtered out tracks with meaningless sequence of panel states (for instance the sequence of $ES : 2 \rightarrow 1 \rightarrow 0$) and deleted trips with length less than 10 m.

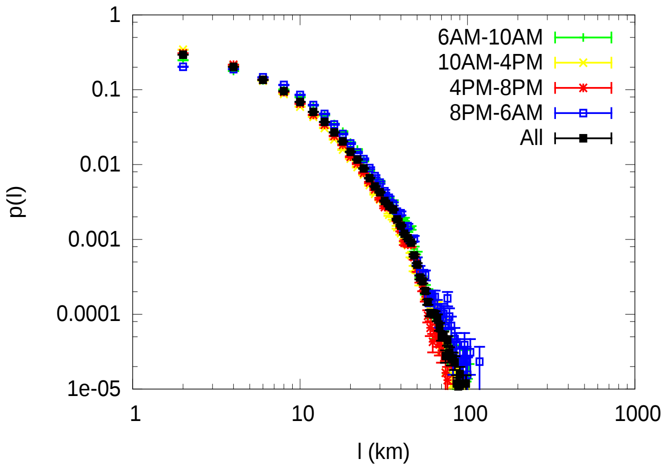

Figure A: Distributions of the length of the GPS tracks (black dots) and the same distribution in the four time slots, from 6AM to 10AM (morning), from 10AM to 4PM (early afternoon), from 4PM to 8PM (evening) and from 8PM to 6AM of the next day (night).

B Hourly dependence

Besides the topology of the urban road network, traffic conditions also affect the time of travel. In order to have a more homogeneous sampling of routes, we divide the overall day into four time slots [3], i.e., from 6AM to 10AM (morning), from 10AM to 4PM (early afternoon), from 4PM to 8PM (evening) and from 8PM to 6AM of the next day (night). Figure . . . shows the distributions of the length of the sampled routes in each one of this time-frames, together with a comparison with the overall distribution of lengths during the whole day. No significant differences were found in these distributions. The morning and evening slots are characteristic of rush hours. We find that, irrespective of the time slot h analyzed, each measured frequency distribution $p_h(t|l)$ of travel time t taken at fixed travel length l is compatible with a log-normal distribution as in the fully aggregated case discussed in the main text. During rush hours travel times are in average larger with respect to those measured during more quiet traffic conditions so that the curves corresponding to the morning and evening slots shift toward the right as shown in Figure B. We can write:

$$p_h(t|l) = \frac{1}{t\sqrt{2\pi\sigma^2_{h,l}}} \exp \frac{-(\log t - \mu_{h,l})^2}{2\sigma^2_{h,l}} \tag{1}$$

where $\mu_{h,l}$ and $\sigma^2_{h,l}$ are respectively the average and variance of the variable $\log t$ at fixed l in the time slot h. From Fig. C we deduce that $\mu_{h,l}$ and $\sigma_{h,l}$ can be expressed, similarly to the aggregated dataset shown in the main, text as:

$$\mu_{h,l} = \alpha_h \log(l/l_0), \qquad \sigma_{h,l} = \sqrt{\sigma^2_{h,l}} = s_h, \tag{2}$$

where the second equation holds for $l > 1\,\text{km}$. The values of the parameters α_h and s_h depend on the time slot considered (see Table A). While the parameter α_h substantially depends on the time slot, being larger in case of congested traffic (meaning that the average time of travel $\langle t_{h,l}\rangle \approx l_h^\alpha$ is higher and the average driving velocity $\langle v_{h,l}\rangle \approx l^{1-\alpha_h}$ is lower), the value of s_h stays practically constant to a value s. This allows a scaling collapse of the $p_h(l|t)$, irrespective of l and h by considering the rescaled variable $\tau = t/\langle t_{h,l}\rangle$ and $s = s_h$ (Fig. D). The universal curve implies the existence of a universal navigation mechanism on the road network not depending on the hour of the day and thus not depending on the traffic situation.

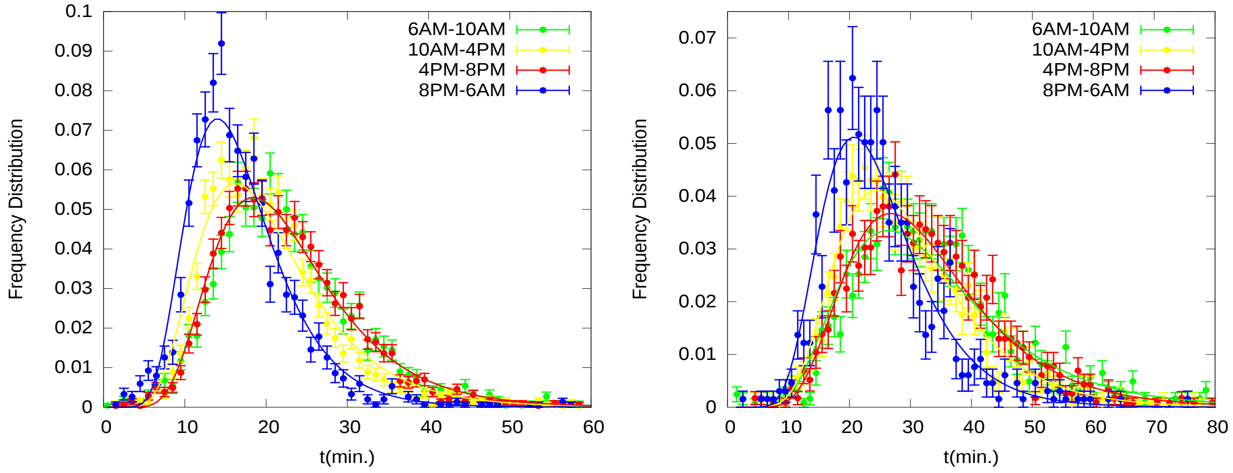

Figure B: Experimental frequency distributions $p_h(t|l)$ of travel time calculated at $l = 7\,\mathrm{km}$ (left) and $l = 12\,\mathrm{km}$ (right) for different time slots h. Solid lines corresponds to log-normal distribution fitted on the data.

time slot		α_h	s_h
6am – 10am	morning	$0.670(4)$	$0.40(9)$
10am – 4pm	early afternoon	$0.633(4)$	$0.40(5)$
4pm – 8pm	evening	$0.666(4)$	$0.37(3)$
8pm – 6am	night	$0.616(3)$	$0.39(7)$

Table A: Dependence of the value of α_h and s_h on the time slot. The larger the α_h value, the more congested is the traffic condition.

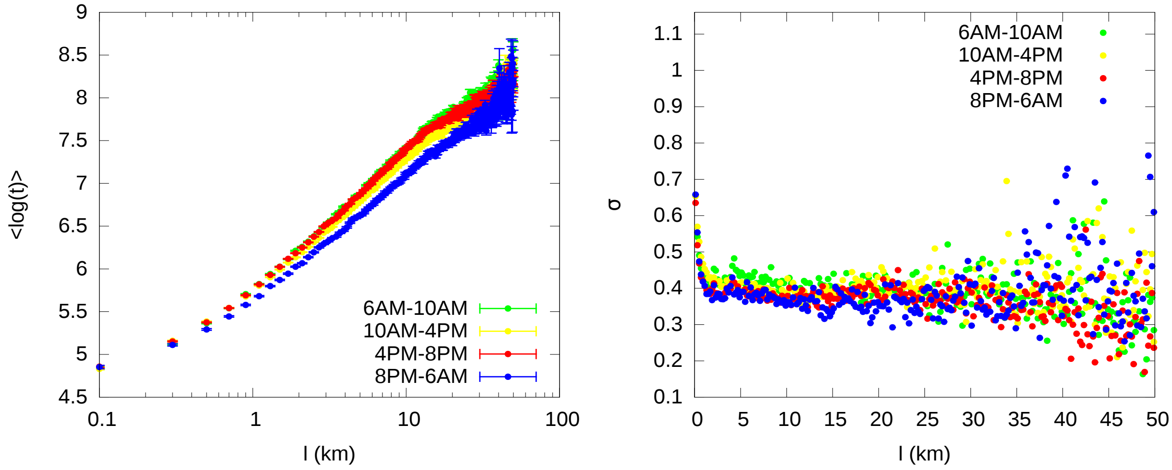

Figure C: (Left panel) Dependence of the parameter $\mu_{h,l}$ on l for different time slots h. The logarithmic scale of the x axis suggests the functional relation $\mu_{h,l} \propto \log l$. (Right panel) Dependence of the parameter $\sigma_{h,l}$ on l for different time slots h. After $1\,\mathrm{km}$ the values of $\sigma_{h,l}$ fluctuate around a constant value $s \simeq 0.40$.

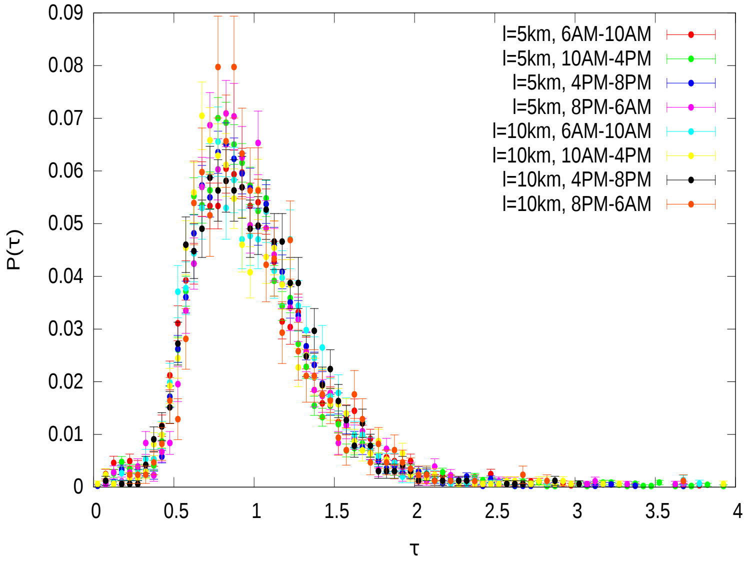

Figure D: Scaling collapse of the estimated probability of traveling a fixed length l at different time slots of the day.

C Time-Space Relation

In this section we show the dependence of travel length l with respect to a fixed travel time t. We estimate the conditional probability $p(l|t)$ with the same procedure used for $p(t|l)$. The right panel of Fig. E shows this distribution for some values of t. Also $p(l|t)$ can be described by a log-normal distribution, though its scaling properties are different. In fact, the average travel length $\langle l_t \rangle$ depends linearly on t (Fig. E left panel). This asymmetry of behavior suggests that car drivers optimize the travel time rather than traveled space. Conversely, we would have seen a super-linear relation between the average travel length and t, corresponding to the sub-linear relation of the average travel time and l presented in the main text.

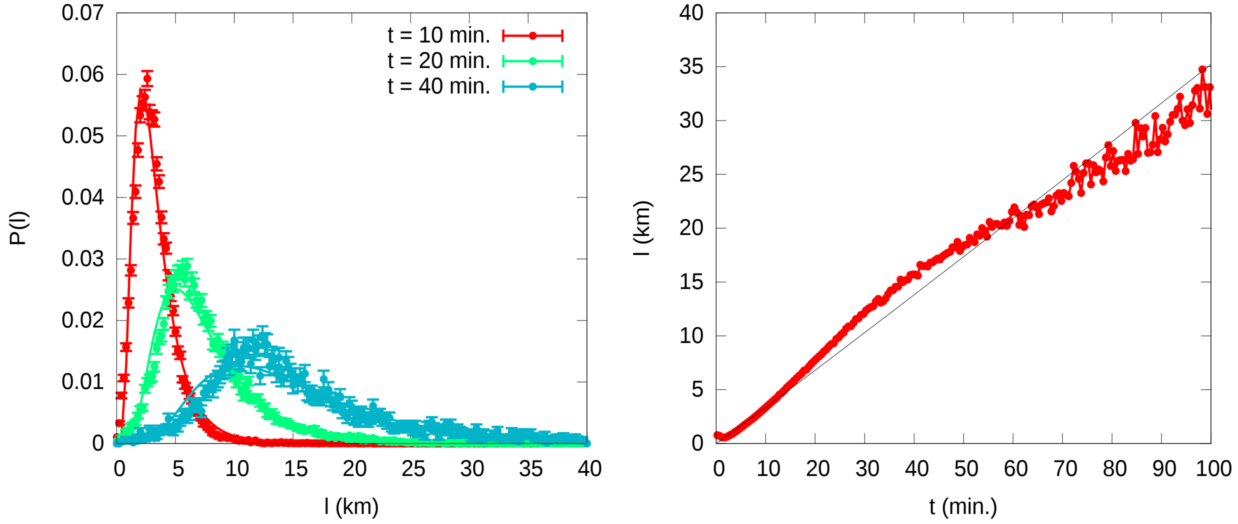

Figure E: (Left) Estimated conditional probabilities $p(l|t)$ for three different values of travel time t. Continuous curves are the corresponding log-normal distributions. (Right) Average travel length $\langle l_t \rangle$ as a function of travel time. The black line shows the linear dependence.

D Model Details and Sampling

The simplest strategy used to build the travel path on the network from a node A to a node B is to choose the shortest path weighting each link with its average travel time. As explained in the main text, this leads to the observed power-law growth of the average travel time with the distance, but on the other hand produces decaying fluctuations around the average, which is in contrast to our experimental results. To heal this unrealistic behavior we have to introduce some sort of noise in the system so to better mimic the actual behavior of drivers. Large fluctuations in short trips and small fluctuations in long trips suggest that short trips might contribute in the construction of large trips so that drivers optimize their paths subdividing them in few intermediate steps. The time optimization is then performed from one step to the other, until the destination is reached (Fig. F).

Assuming that a driver has to travel from the node A to the node B in the network, we choose an optimization distance $l_{\rm optim}$ from a uniform distribution in $[3, l(A,B)]$ where $l(A,B)$ is the euclidean distance between A and B. Starting from A, an intermediate n_1 step is chosen within a radius $l_{\rm optim}$ from A so that the angle between the lines connecting A and n_1 and A and B is smaller than an assigned value α ($\alpha = \pi/6$ rad in the following). The path between A and n_1 is chosen by minimizing the traveling time. The process is repeated from n_1 to the next intermediate step n_2 and so on, until the distance between a generic step n_i and B is less than $l_{\rm optim}$. After that, $n_{i+1} = B$ and the process ends. In our model, the average $\langle t \rangle$ goes as l^α as in agreement with the observed behavior. In Fig. G we show how the exponent α changes with the fast link density (horizontal axis) and the different velocity with which they can be

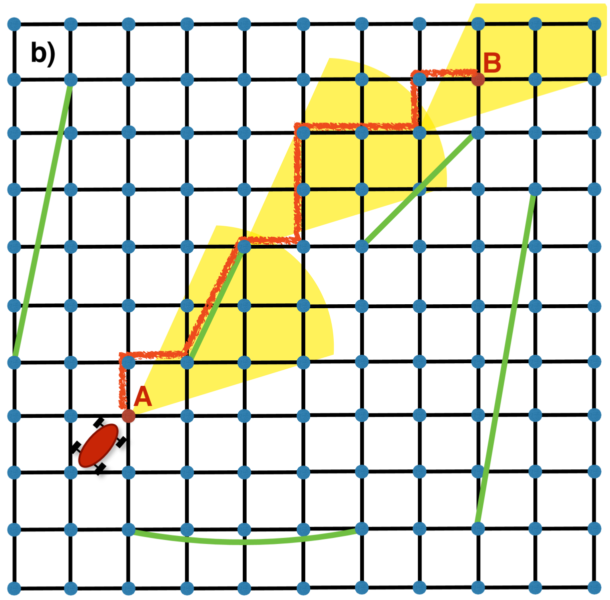

Figure F: Sketch of the stepwise algorithm used to connect the starting point A to the final point B. The yellow sectors represent the areas in which the driver can choose his next step. The fast link just below point B would be convenient to travel, but it falls outside the yellow sectors and therefore is out of reach.

traveled. When no fast links are present, the exponent α is equal to one since all links are traveled with unit velocity. The exponent α is unity also when all nodes in the grid are connected by fast links with the same average velocity. This means that our model is characterized by an optimal density of fast links, in correspondence of which α is minimum and the average travel velocity gets its maximum.

A further interesting property of our model is the power-law behavior connecting the number of nodes that can be covered in a fixed travel time t, i.e., $N_t \propto t^{\delta}$. In case no fast links are present, one trivially gets $\delta = 2$. When fast link are added, we measure the exponent δ as the slope (in double logarithmic scale) at the inflection point of the cumulative number of isochronous paths N_t (as in Ref. [1]) as depicted in Fig H.b.

The behavior of δ as a function of the number of fast links is shown in Fig. H.a, where different colors stand for different fast link velocities. The value of δ stays larger than 2.0 with small values of $N_{\mathrm{shortcuts}}$ and grows until it reaches a maximum around 2.8 depending on fast link velocity v, similarly to what has been found for real urban networks [1,5]. When the number of fast links reaches its maximum value, the value of δ

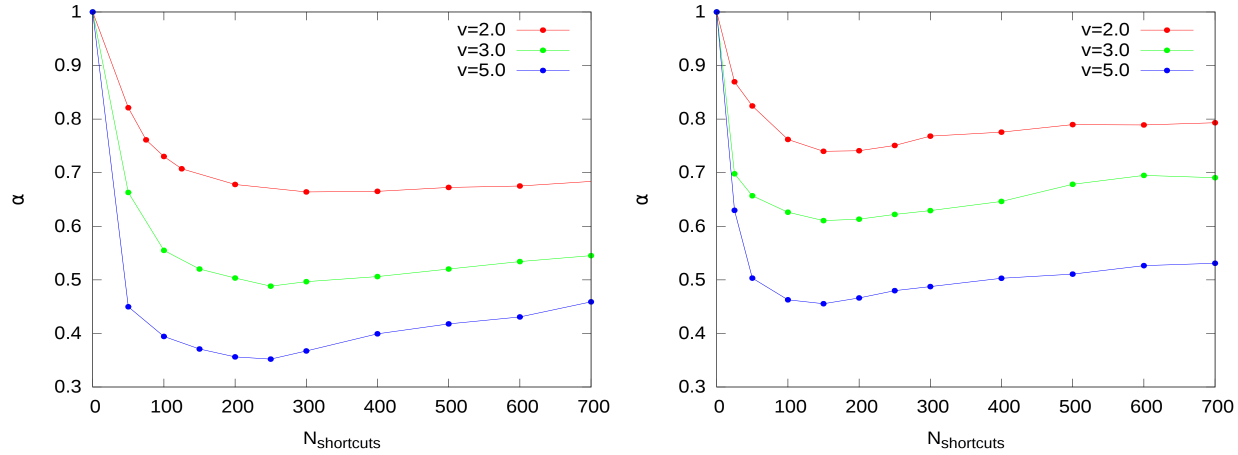

Figure G: In our model, the average $\langle t \rangle$ goes as l^{α}. Here we show the dependence of α on the number of shortcuts (horizontal axis) and on the average velocity with which they can be traveled (different colors). The chosen grid contains $N = 100 \times 100$ nodes and the velocity on simple links is set to unity. The left panel refers to our model with *global* time travel minimization, while the panel on the right is obtained by a *stepwise* optimization of the travel time. In case $v = 1$, the exponent α would be identically equal to unity, irrespective of the fast link density. When the number of fast links gets its maximum value, the exponent α reaches the unit value since there exists a direct fast connection from the initial to the final destination, traveled at constant velocity.

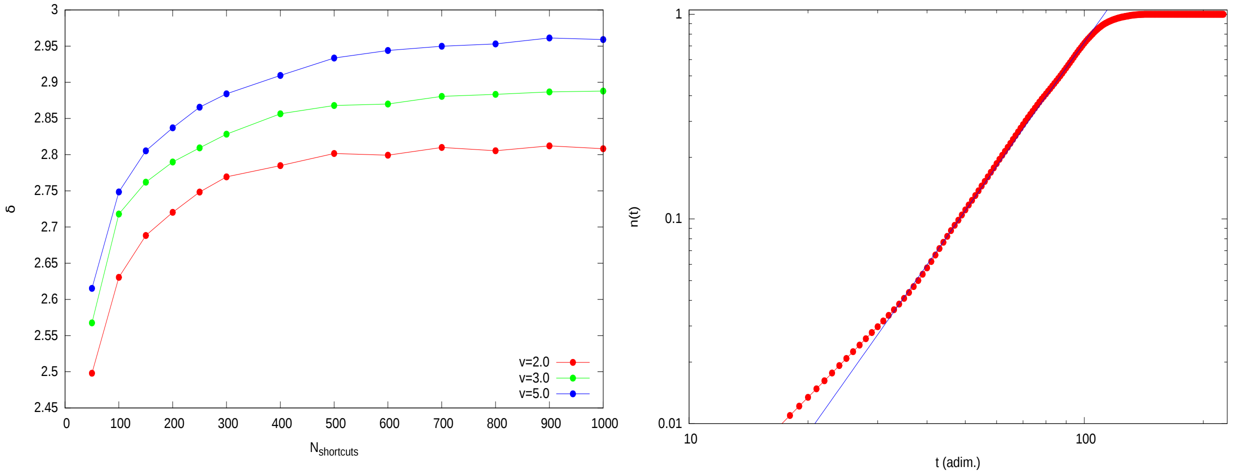

Figure H: (Left) Dimension of the network computed as the slope of N_t at its inflection point as a function of the number of shortcuts in a 100×100 grid, for three different fast link velocities. When the number of fast links is sufficiently high, the curves start to decrease eventually reaching the limit value of 2. (Right) Cumulative number of isochronous path N_t as a function of the time t for a 250×250 grid with 100 shortcuts and shortcut velocity equal to 2. The blue line is the slope at the inflection point of the curve. This dimension has been measured for some Urban Networks in Germany [5]. The dimension varied from one city to another ranging from a minimum value of about 2.0 to a maximum value of ca. 2.7.

E Grid model with realistic speed distribution

In our basic model the average velocities of links are trivially chosen to be constant, i.e., unit velocity on the normal grid links and $v > 1$ on fast links. This is sufficient to reproduce most of the observed behavior apart the specific form of the function $p(t|l)$ that in the simple model is not a log-normal (see Fig. 5 in the main text). To refine the model, we would like to define the average velocities on links by extracting them from the distribution of actual velocities on urban roads, still allowing model drivers to perform the optimal local search depicted in Fig. F. Moreover, in this way we fix the otherwise arbitrary ratio between normal and fast link velocities.

The set of considered velocities stems from an integration of both OpenStreetMap and Google Maps databases (for sake of simplicity in the following we will refer to the network built with this data as the *GoogleMaps Network*) [4]. The GoogleMaps Network dataset consists of a list of roads whose ends (crossroads) are identified by their coordinates, with each road characterized by an average traveling time. We infer the coordinates of the crossroads and their connections from OpenStreetMap [9], and feed the Google Directions API [10] with them in order to extract the average traveling time of each road. We considered the GoogleMaps Network for part of the center of the cities of Rome (23504 links) and London (112377 links).

In Fig. I.a we show the frequency distribution $p(v)$ of the average velocities in the GoogleMaps Network of Rome, where the velocities have been scaled so that their average is set to unity. This distribution is peaked around $v = 1$ and is right skewed. We argue that the velocities much larger than the average characterize arterial roads, which are, at least in principle, traveled at a higher speed than that of regular streets. To assign average velocities to the links of our $L \times L$ grid model with shortcuts, we proceed as follows. We fix the number of shortcuts, i.e. fast links, $N_{\mathrm{shortcuts}}$ and calculate the fraction of regular links as $f = \frac{2L(L-1)}{N_{\mathrm{shortcuts}}+2L(L-1)}$, where $2L(L-1)$ is the number of links in the grid. We define $v_p(f)$ as the fth-percentile of the distribution of the velocities, i.e., $f = \int_{v_p(f)}^{\infty} p(v)dv$ (see Fig. I.b) and assign to normal links an average velocity extracted from $p(v)$ with $v < v_p(f)$ and to fast links a velocity extracted again from $p(v)$ but with $v \geq v_p(f)$. For this variation of the model we still assume that drivers minimize the time of travel by fixing intermediate steps.

In Fig. J we synthesize the results of this choice of link velocities. We still recover a power-law dependence of $\langle \log t \rangle$ (Fig. J.a), a constant behavior of the variance σ_l^2 of $\log t_l$ at large trip lengths (Fig. J.b), and an estimated scaling collapsed distribution $p(\tau)$ not well represented by a log-normal distribution (Fig. J.c). On the contrary, the behavior of the variance of $\log t_l$ is more similar to the real case for short trips. In fact, in the case of the basic simple model with two velocities, the standard deviation σ_l increases from zero to the expected constant value, as shown in Fig. J.b with the green curve, while in the real measured case the same quantity is decreasing (Fig. 1.d of the main text). This is because for short trip lengths l in our basic model, it is unlikely to travel through a fast link so that the regular Manhattan paths are the most used and these are traveled at the same constant unity velocity. In the actual case, instead, the noise induced by the different average velocities of streets, street regulations and traffic conditions affect the variability of the time of travel of short trips more than long ones. The introduction of stochastic velocities extracted from a realistic distribution partially reproduces this behavior (red curve of Fig. J.b). With respect to the actual measured σ_l, which decreases monotonically toward a constant value, there is here the appearance of a deep minimum. There is no surprise that the behavior of our model does not mirror faithfully the real situation. In fact, our model displays an oversimplified topology of an urban road network and the velocities on its links are extracted independently from a realistic distribution thus lacking any sort of correlations. In the following section we shall deal with these two inconsistencies.

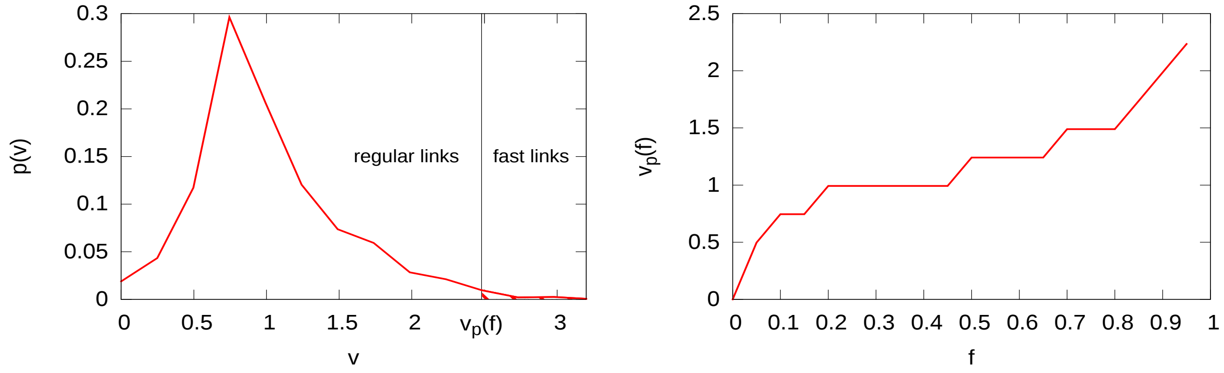

Figure I: (Left) Frequency distribution of the velocities $p(v)$ for the GoogleMaps Network of Rome. The vertical line indicates the values of $v_p(f)$ for $N_{\text{shortcuts}} = 700$ in a 100×100 grid. The velocities of the shortcuts are taken from the filled part of the distribution right of the vertical line $v_p(f)$. Velocities on the horizontal axis have been rescaled by the first moment of the distribution, which equals $\sim 20\,\text{km/h}$. (Right) $v_p(f)$ as a function of the fraction of regular slow links f for the GoogleMaps Rome Urban Network.

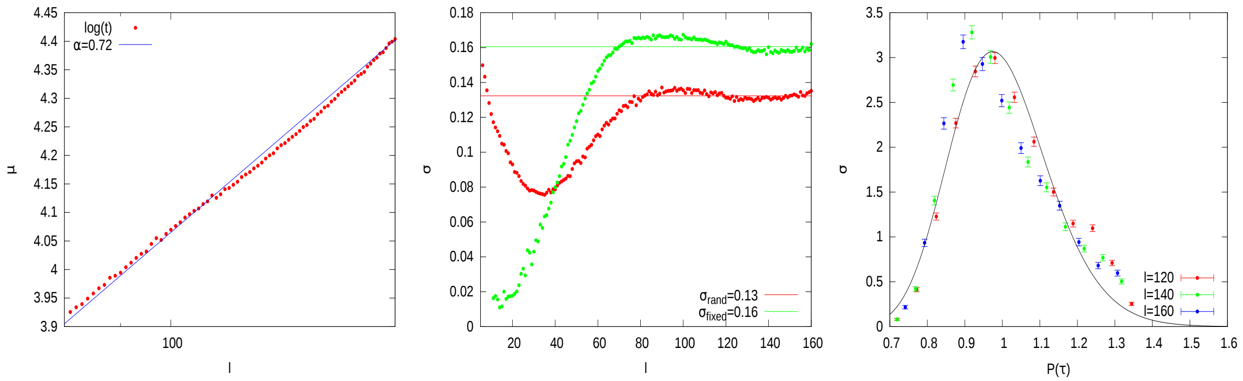

Figure J: (Left) Dependence of the mean value $\log t_l$ of the logarithm of travel time t by fixing the travel length l. Points have been obtained by stepwise optimizing the time of travel on a 100×100 grid with $N_{\text{shorcuts}} = 100$ and a realistic velocity distribution sampled from the GoogleMaps Network of Rome. In this case we have $f = 0.995$ and $v_p(f) = 3.30$. (Center) Dependence of the std deviation of $\log t_l$ for the same grid with realistic velocity distribution (red) and with fixed velocities with $v = 3$ on the fast links (green). (Right) Collapse of the distribution $p(t|l)$ using the scaling property explained in the main text.

F Navigation algorithm in the Rome and London urban networks

In this section we further refine our model by considering the actual road network of Rome and London. The average velocities of roads are those extracted from the combined information of OpenStreetMap and Google Directions introduced in the previous section. In this way we are considering both a realistic topology of road connections and their actual average time travel with the correlations between adjacent roads included.

We apply the navigation algorithm with time optimization and intermediate steps after sampling random origin and destination points over the network. We obtain as before that the average of the logarithm of the travel time $\log t_l$ grows with the length of the path l as a power-law (Fig. K) with an exponent higher than the one found in real data. The fluctuations of $\log t_l$ decrease with l for short trips and tend to a constant as l grows. The value of this constant is lower than the one found in real data (Fig. K top panels). The distributions $p(t|l)$ have the same scaling property of those found in real data (Fig. K bottom panels) so that an universal collapsed function can be obtained. This function cannot be a gaussian function since it shows a significant skewness value. Interestingly, contrarily to what found in the case of our grid model with simple topology, the Kolmogorov-Smirnov test does not exclude that these distributions are log-normal. This suggests that velocity correlations between adjacent roads are an important ingredient of the model.

Besides the urban network of Rome, all previous measures have been repeated on the urban network of London, built with the same procedure used for Rome. The network of London contains $106,352$ nodes and $112,377$ links, while the network of Rome has $19,367$ nodes and $23,504$ links. Fig. L displays the analysis performed in the case of London. All previous results are confirmed, including the scaling behavior of $p(t|l)$ with the exponent of the power law relation between $\log t_l$ and l as well as the constant value of its variance σ_l^2 being similar in the two networks.

The scaling property and the qualitative behavior of the distribution $p(t|l)$ is captured by our model, although the optimization process is less efficient than the one occurring in real life and the fluctuation are dumped in some way. Notice that the measures obtained with GPS data are aggregated at the end of the trip of each driver, meaning that during her travel the trajectory of the car had been affected by all the dynamical fluctuations of speed due to the traffic situation at the time the trip occurred. On the other hand, when sampling paths on a network like the GoogleMaps one, these fluctuations are eliminated by the averaging process done by Google over the links of the network. In this way fluctuations due to traffic are averaged together in the average travel speed assigned to every road. A more predictive and accurate model might consider the interaction between different paths converging to the same roads at the same time and their effect on the traveling speed.

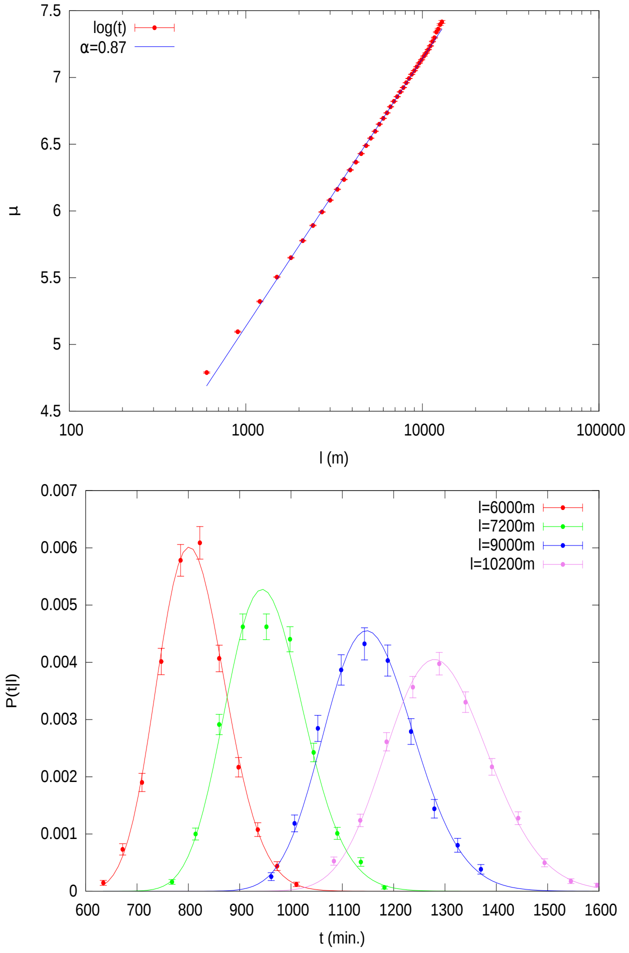

Figure K: This figure can be found in the main text, too (Fig. 6); we duplicate it also here for convenience. (Top Left) Dependence of the mean value $\langle \log t \rangle$ of the logarithm of travel time t upon travel length l. These points were obtained by optimizing the time of travel with intermediate steps on the Rome urban network (GoogleMaps network). (Top Right) Standard deviation σ_l of $\log t$. The paths of these figures are sampled using the navigation algorithm with intermediate steps and a sampling angle of 30° (red points) and using the shortest path (global optimization) from the origin to the destination (green points). (Bottom Left) Distributions $p(t|l)$ for some selected values of l. Continuous lines are log-normal approximations of the distributions. (Bottom Right) Collapse of the same distribution using the scaling property derived in the main text. The black curve is the distribution of the scaled variable $\tau = t/\langle t \rangle$.

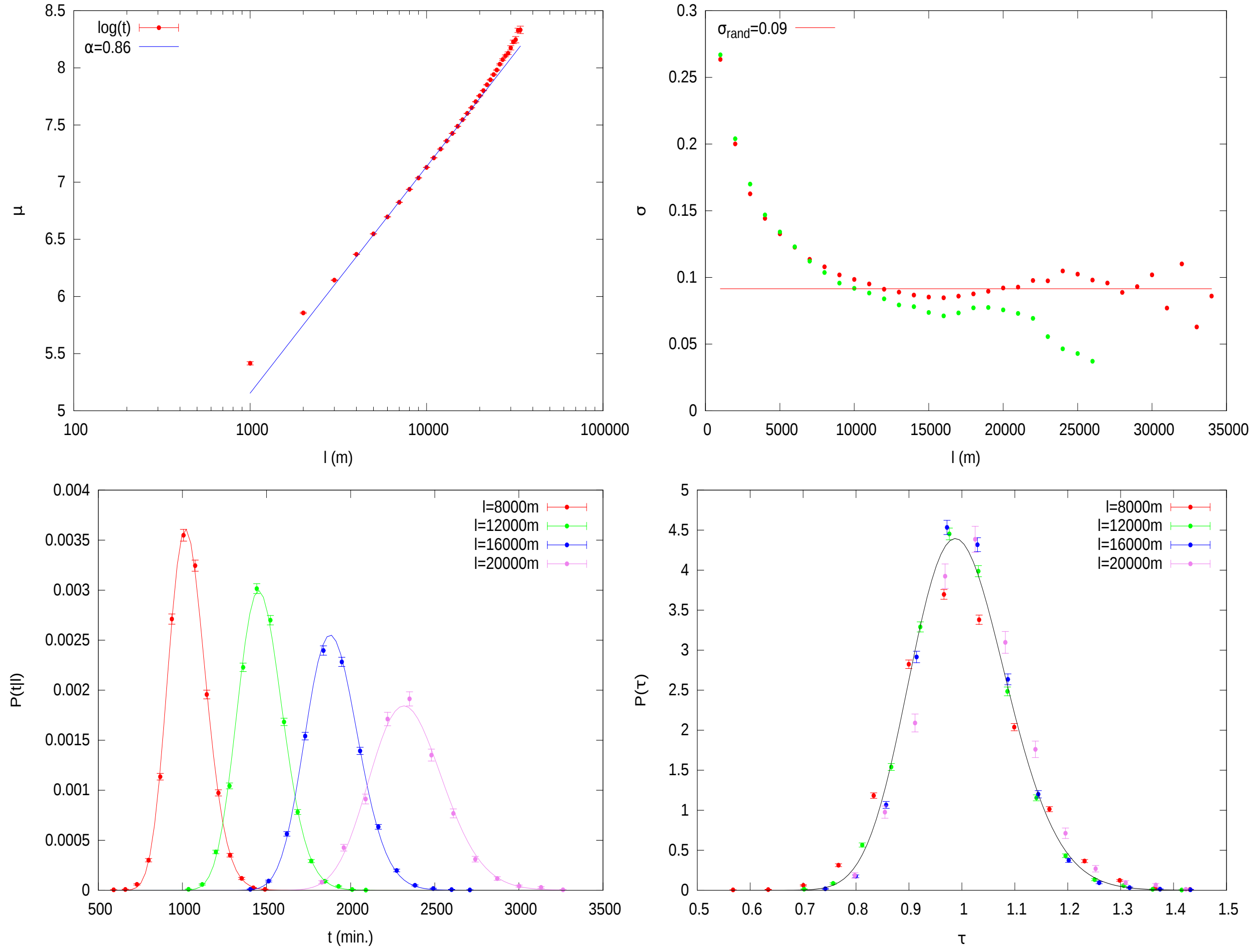

Figure L: (Top Left) Dependence of the mean value $\langle \log t \rangle$ of the logarithm of travel time t upon travel length l. These points were obtained by optimizing the time of travel with intermediate steps on the London urban network (GoogleMaps network). (Top Right) Standard deviation σ_l of $\log t$. The paths of these figures are sampled using the navigation algorithm with intermediate steps and a sampling angle of 30° (red points) and using the shortest path (global optimization) from the origin to the destination (green points). (Bottom Left) Distributions $p(t|l)$ for some selected values of l. Continuous lines are log-normal approximations of the distributions. (Bottom Right) Collapse of the same distribution using the scaling property derived in the main text. The black curve is the distribution of the scaled variable $\tau = t/\langle t \rangle$.

G Log-normal distribution and multiplicative process

The fact that the distributions $p(t|l)$ in Fig. K and Fig. L can be described by log-normal distributions suggests that some kind of multiplicative process might be in action. First of all, we notice that since $p(t|l)$ are log-normal also the distributions $p(v|l)$, where $v = l/t$ have to obey a log-normal distribution. In fact, it is easy to see that

$$p(v|l) = p(t|l)\left|\frac{dt}{dv}\right| = \frac{1}{v\sqrt{2\pi\sigma_l^2}}\exp\frac{-(\log v - \log l + \mu_l)^2}{2\sigma_l^2}, \tag{3}$$

where μ_l and σ_l are the parameters of $p(t|l)$. Thus it is possible to suppose that the multiplicative process occurs on the average velocities rather than on the total travel time.

A path Π of length l can be identified by the series of crossing times t_k and traveled space l_k at each node, i.e., $\Pi = \{(l_1, t_1), (l_2, t_2), \ldots, (l_{m-1}, t_{m-1}), (l, t)\}$ where t is the total travel time. Similarly it is possible to define the average velocity at the kth step as $v_k = l_k/t_k$. Following the results of Refs. [3, 7] for the spreading of novelties, a multiplicative process that leads to a log-normal distribution of variables can be defined as

$$v_{k+1} = e^{f(k)}(1 + g(k)X_k)\, v_k \simeq v_k\, e^{f(k)+g(k)X_k} \tag{4}$$

where $f(k)$ is so that $\sum_{k=0}^{\infty} f(k)$ and $\sum_{k=0}^{\infty} g(k)$ exist, X_k are i.i.d. with zero mean and unit variance. The last approximation holds whenever the $g(k)$ are small (i.e. they are close to 0). In this case, defining $\gamma_k = \log(v_k)$, we have

$$\gamma_{k+1} - \gamma_0 = \sum_{i=0}^{k} f(i) + g(i)X_i, \tag{5}$$

and the distribution of the sum $\sum_{i=0}^{\infty} g(i)X_i$ converges to a normal distribution [8].

Considering a set of paths of length l, sampled on the GoogleMaps network with the intermediate step navigation algorithm, we can estimate $\widetilde{X}_k = \gamma_{k+1} - \gamma_k$. For completeness we report that the variables $\widetilde{X}_k$ result to be weakly correlated with a correlation coefficient of 0.1 and p-value of 0, and not identically distributed. We compute $f(k) = \langle\widetilde{X}_k\rangle$ and $g(k) = \mathit{Var}[\widetilde{X}_k]$ at every step of a path. From the analysis of the behavior of these quantities, shown in Fig. M, these functions can be well described by

$$f(k) = Ae^{-k/k_0} \quad \text{and} \quad g(k) = B(1+k)^{-b}e^{-k/k_1}, \tag{6}$$

where $A = 0.015$, $k_0 = 13.9$, $B = 0.41$, $b = 0.83$ and $k_1 = 390$. It is easy to check that with these parameters the series are both convergent and $g(k)$ is small for $k > 0$.

The behavior of these two functions can be understood by realizing that as the step of the path increases we get a convergence to the average process. In fact $f(k)$ decreases rapidly to zero so that the ratio of the average velocities on subsequent links oscillates around one. This indicates that v_k converges after few steps to a value close to its average and then oscillates around this value. The expression of $g(k)$ indicates that these oscillations decrease until they approach zero value for large k, when v_k is very close to the real average value. In order to make the rhs of Eq. (5) converge to a normal distribution, the variables X_k must be i.i.d.. We compute them by defining

$$X_k = \frac{\widetilde{X}_k - f(k)}{g(k)}. \tag{7}$$

Fig. N shows the distributions of X_k for some values of k. All the variables are identically distributed and uncorrelated (almost vanishing correlation coefficient). The hypotheses of Refs. [3, 7] are satisfied and the resulting distribution of the average velocities tends to a log-normal.

To highlight the differences, the previous reasoning can be repeated on the grid model with shortcuts, where the final distributions $p(t|l)$ are not log-normal to understand which of the hypoteses of Refs. [3, 7] do not hold. Since the convergence of $f(k)$ and $g(k)$ is assured by the convergence of v_k to an average value, the only possibility is that the variables X_k are not identically distributed or are not uncorrelated. The latter hypothesis is easily confuted by looking at the correlation coefficients between X_k and X_{k+1} which is always zero for every k, so that the X_k are

not correlated. On the other hand it can be easily checked that they are not identically distributed by computing the cumulants of degree larger than 2 (Fig. O). In fact, these cumulants have a clear dependence on the step k on the grid model, thus signaling that they obey different statistics, while on the Rome Urban Network they oscillate around a constant value. Although we have shown that a sort of multiplicative effect is in action in the system, we could not find a satisfactory explanation to its emergence.

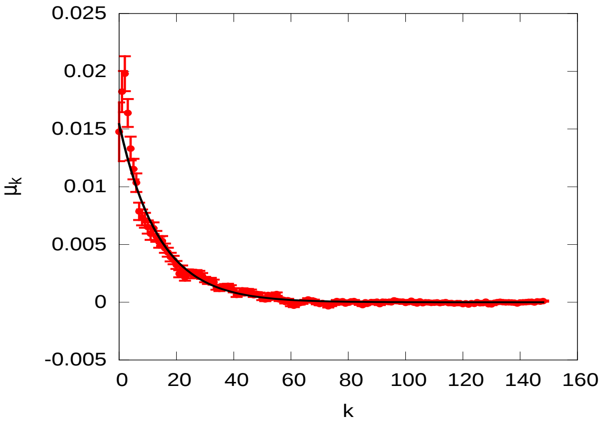

Figure M: $\langle \widetilde{X}_k \rangle$ (left) and $\sqrt{Var[\widetilde{X}_k]}$ as functions of k for the paths with $l = 9000\,\text{m}$ on the GoogleMaps Network of Rome. Continuous lines are fits of the functions $f(k)$ and $g(k)$ of Eq. (6).

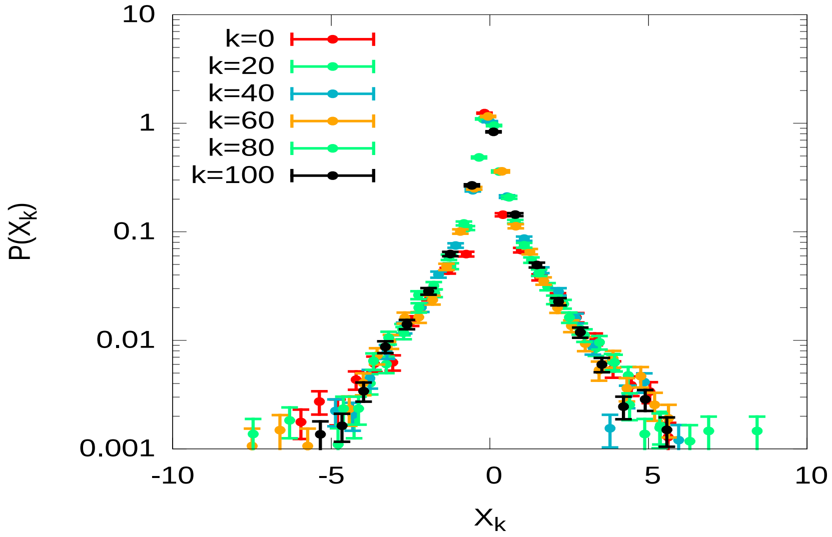

Figure N: Distributions of the variables X_k defined in Eq. (7) for some values of k. It is evident that the X_k are extracted from the same distribution.

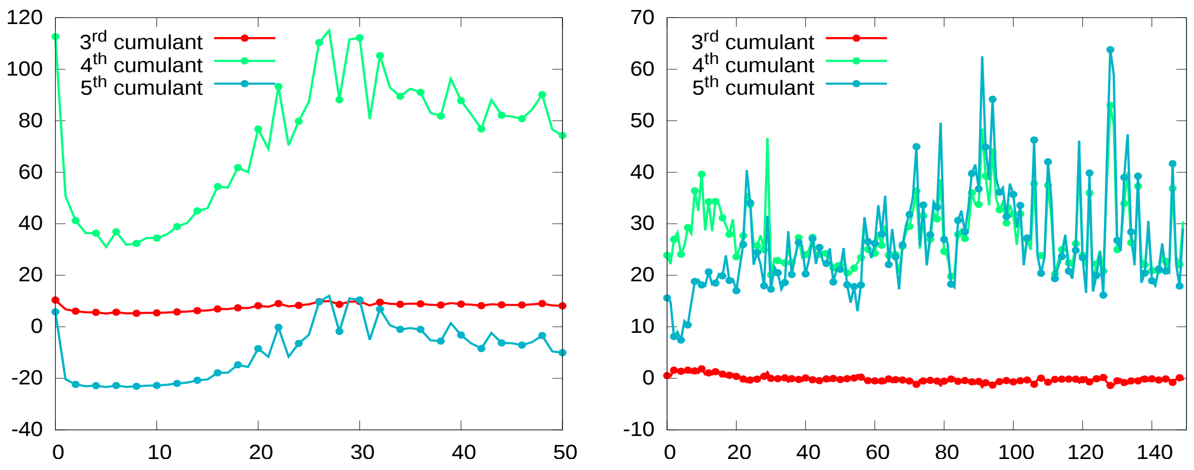

Figure O: Third, fourth and fifth cumulants of the distributions of X_k as k varies. Left panel corresponds to the measures performed over the grid model ($L = 100$, $v = 3$ and paths of length $l = 100$); right panel corresponds to measures performed over the Rome Urban Network (paths of length $l = 9000\,\mathrm{m}$).

References

[1] Brockmann, D. *et al.* The scaling laws of Human Travel. *Nature* **439**, (January 2006).

[2] Gallotti, R. Statistical Physics and Modelling of Human Mobility, *PhD Thesis, Physics Department, University of Bologna*, (2013).

[3] Wang, P. *et al.* Understanding Road Usage Pattern in Urban Areas. *Sci Rep.* **2**, (2012).

[4] Alina Sîrbu, Saverio Caminiti, Pietro Gravino, Vittorio Loreto, Vito D.P. Servedio and Francesca Tria, A new platform for Human Computation and its application to the analysis of driving behaviour in response to traffic information. submitted to *CCS14 Proceedings in Human Computation*

[5] Lammer, S. *et al.* Scaling laws in the spatial structure of urban road networks.*Physica A* **363**(1), 88-95 (2006).

[6] Fang, Wu *et al.* ”Novelty and collective attention.” *Proceedings of the National Academy of Sciences* 104.45, 17599-17601 (2007).

[7] Yan, K. K., & Gerstein, M. “The spread of scientific information: insights from the web usage statistics in PLoS article-level metrics” *PloS one*, 6(5) (2011).

[8] Embrechts, P., & Makoto, M. ”The central limit theorem for summability methods of iid random variables.” *Probability Theory and Related Fields* 68.2,191-204 (1984)

[9] OpenStreetMap data export. `http://www.openstreetmap.org/export`. Accessed: 2014-10-16.

[10] Google Directions API. `https://developers.google.com/maps/documentation/directions/`. Accessed: 2014-10-03.
